# Coronary artery lesion distribution in patients with chronic kidney disease undergoing percutaneous coronary intervention

**DOI:** 10.1080/0886022X.2022.2093748

**Published:** 2022-07-08

**Authors:** Naofumi Ikeda, Toshihide Hayashi, Shikou Gen, Nobuhiko Joki, Kazuhiko Aramaki

**Affiliations:** a Department of Nephrology, Saitama Sekishinkai Hospital, Sayama, Japan; b Division of Nephrology, Toho University Ohashi Medical Center, Tokyo, Japan; c Department of Cardiology, Saitama Sekishinkai Hospital, Sayama, Japan

**Keywords:** Coronary atherosclerosis, mechanical force, location, distribution

## Abstract

**Purpose:**

To determine the location of coronary atherosclerosis distribution observed in patients with chronic kidney disease (CKD).

**Methods:**

A cross-sectional study was conducted using the database of cardiovascular medicine data from Saitama Sekishinkai Hospital to clarify the association between renal function and angiographic characteristics of coronary atherosclerosis. In total, 3268 patients who underwent percutaneous coronary intervention were included. Propensity score matching revised the total to 1772. The association of renal function with the location and/or distribution of coronary atherosclerosis lesions was then examined.

**Results:**

Overall, coronary lesion was observed in the left anterior descending coronary artery (LAD) in 56% patients, whereas 28% and 22% were in the right coronary artery (RCA) and left circumflex coronary artery (LCX), respectively. LAD was most affected and observed in 57% patients with stage 1 CKD. RCA was second-most affected, at 26% CKD stage 1, but it increased to 31%, 38%, and 59% in CKD 3, 4, and 5, respectively. In CKD 5 patients, the RCA was the most affected artery (59%), with 41% LAD lesions. Logistic regression analysis after propensity score matching showed that the odds ratios for an RCA lesion was 3.658 in CKD 5 (*p* = .025) compared with CKD 1 after adjusting for traditional risk factors.

**Conclusion:**

The prevalence of RCA lesions, but not LAD or LCX lesions, increased with increasing CKD stage. The pathophysiology of coronary atherosclerosis may differ by lesion location. Deterioration of renal function may affect progression of atherosclerosis more in the RCA than in the LAD or LCX.

## Introduction

It has been almost 20 years since the American Heart Association declared that chronic kidney disease (CKD) is recognized as a condition associated with major risks for the development of cardiovascular disease [1]. In particular, coronary atherosclerosis has a great impact on prognosis and is of considerable clinical importance in CKD patients [2]. Although the management of coronary artery disease (CAD) in CKD patients is changing and has improved during this period [3], cardiovascular disease remains the leading cause of morbidity and mortality in patients with end-stage kidney disease (ESKD) [4]. Multivessel diseases and multiple complex calcified lesions are CAD characteristics, which are often refractory to coronary revascularization therapy and lead to a poor prognosis in ESKD patients [5]. Preventive drug therapy for coronary atherosclerosis, such as statins, also has limited effectiveness [6], partially due to the accumulation of nonclassical coronary risk factors. Therefore, a new basis for understanding the pathophysiology of CAD in these patients may be needed to improve patient prognosis.

Several recent studies have suggested a difference in the distribution of CAD between the general population and CKD population. Culprit lesions for acute coronary syndrome are found more often in the left anterior descending coronary artery (LAD) than in the right coronary artery (RCA) and left circumflex coronary artery (LCX) in the non-CKD population [7]. In contrast, in CKD patients, 42% of lesions were seen in the RCA, a higher rate than in the LAD (35%) in 1447 primary percutaneous coronary intervention (PCI)-treated acute myocardial infarction patients [8]. By contrast, in non-CKD patients, 50% of lesions were observed in the LAD, a higher rate than in the RCA (33%) [8]. These findings imply that the pathophysiology of coronary atherosclerosis might differ between the two patient groups: those with a preserved glomerular filtration rate (GFR) and those with a reduced GFR. Moreover, more proximal lesion location was seen in CKD patients than in non-CKD patients in a study of 381 acute coronary syndrome patients [9]. This phenomenon was more pronounced in the RCA than in the other two arteries. Compelling evidence leads to the hypothesis that the initiation and progression patterns of coronary atherosclerosis differ in the three major coronary branches and/or the location of the lesion in CKD patients. However, the exact nature of potential differences in lesion location, distribution, and composition are not well classified in patients with CKD, as indicated by a recent review which identified research needs related to pathology regard CKD and CAD [10].

This study aimed to determine (1) whether the distribution of coronary artery lesions shows a tendency to parallel CKD stage development, and if yes, (2) whether lesions in the distribution of the RCA are predominantly seen in CKD patients. Although this was a preliminary study, and it is impossible to clarify the mechanism for our hypothesis, these results may provide a catalyst for understanding the pathophysiology of CAD in the uremic milieu.

## Materials and methods

### Study design and participants

**Figure 1.**
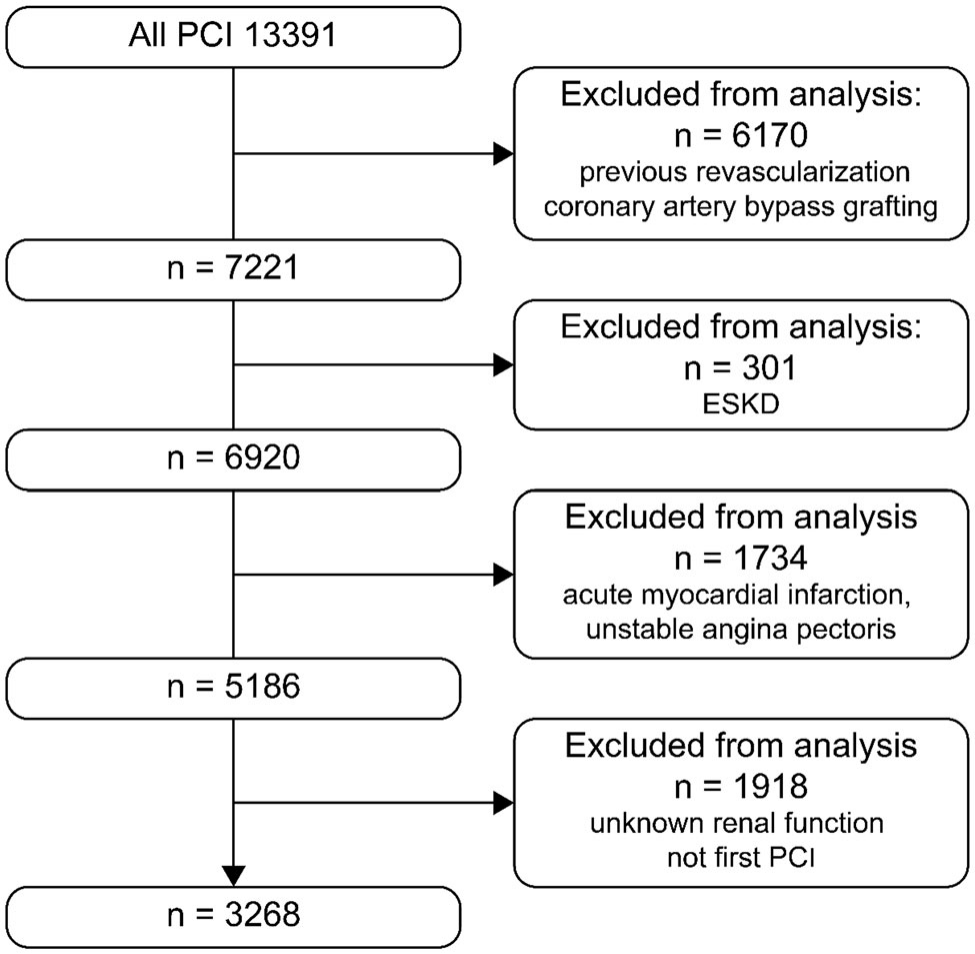
Patient flow chart. PCI: percutaneous coronary intervention; CKD: chronic kidney disease.

A cross-sectional study was conducted using retrospectively collected cardiovascular medicine data from the Saitama Sekishinkai Hospital. Between January 2003 and December 2017, 13,391 patients underwent PCI for myocardial infarction, acute coronary syndrome, and angina pectoris. To specifically examine the effect of renal function on the characteristics of coronary atherosclerosis, the following patients were excluded: (1) patients with previous coronary revascularization; (2) ESKD patients on dialysis therapy; (3) patients with emergent PCI due to acute myocardial infarction, acute coronary syndrome, and unstable angina; and (4) patients with missing renal function data and past PCI. As shown in Figure 1, the number of patients excluded for each criteria 1–4 equaled 6170; 301; 1734; and 1918; respectively. In total, 3268 patients were enrolled in the study. The association of renal function with the angiographic characteristics of coronary atherosclerosis, especially the location and/or distribution of coronary atherosclerosis lesions, was then examined.

### Data collection

The clinical diagnosis of the underlying kidney disease (not necessarily biopsy-proven), history of CAD, and presence of diabetes mellitus, hypertension, and dyslipidemia were extracted from the records. Blood samples were collected at the time of PCI. Whole blood was used to measure hemoglobin (Hb) levels, and other biochemical assays were performed using serum samples. Serum creatinine (Cr), total cholesterol (TC), low-density lipoprotein cholesterol (LDL-C), high-density lipoprotein cholesterol (HDL-C), and brain natriuretic peptide (BNP) levels were measured using routine laboratory methods in the hospital. The non-HDL-C level was calculated by subtracting the HDL-C level from the TC level (non-HDL-C [mg/dL] = TC [mg/dL] − HDL-C [mg/dL]). Diagnosis of dyslipidemia was defined as an LDL of 120 or higher, or the use of statins, or the use of ezetimibe.

### Echocardiographic assessment

Measurements and calculations of the morphology of the heart was recorded from patient records. These measurements include left ventricular mass index (LVMI), ejection fraction (EF), and relative wall thickness (RWT). The measurement of the RWT was determined using the following formula, where T_lvpw_ represents the thickness of the left ventricular posterior wall and D_lved_ represents the diameter of the left ventricle at the end of diastole:

$$(2*T_{\mathrm{lvpw}})/D_{\mathrm{lved}}$$

The LVMI was calculated using the area length method as follows:

$$1.05\{\left[\frac{5A1(a+d+t)}{6}]-[\frac{5A2(a+d)}{6}\right]\}$$

Finally, EF was determined using the biplane modified Simpson method: V = π/4×$\sum_{i=1}^{20}ai\times bi\times (\frac{L}{20})$

### Renal function

The participants were divided into five groups according to the GFR at the time of PCI. Estimated GFR (eGFR) mL/min/1.73 m^2^ was calculated using the equation: 194 × serum Cr − 1.094 × age − 0.287 (× 0.739 in females) [11]. Stages of CKD were based on the following values of eGFR (mL/min/1.73 m^2^): stage 1, 90 ≤ eGFR; stage 2, 60 ≤ eGFR < 90; stage 3, 30 ≤ eGFR < 60; stage 4, 15 ≤ eGFR < 30; and stage 5, eGFR < 15. The distribution of the coronary artery lesion location was compared among the five CKD groups.

### Lesion location

Coronary angiographies were performed in the standard manner, with stenosis of any coronary artery >75% considered significant. In the left main coronary artery (LMT), stenosis >50% was considered significant. The distribution of significant coronary artery stenosis was compared among the five CKD groups. The causative lesion of the CAD was documented by angiography (≥75% stenosis of a major epicardial coronary artery, ≥50% stenosis of the LMT, and classic angina). Responsible physicians made all decisions regarding the PCI procedure. PCI success as seen on angiography was defined as normal coronary artery flow and stenosis <50% of the luminal diameter of the causative lesion.

### Statistical analysis

Data are summarized as numbers, prevalences, arithmetic means ± SD, medians (interquartile range), or tertiles (interquartile range), as appropriate. Comparisons of prevalences and values between groups were performed using analysis of variance and the χ^2^ test. The association between CAD and CKD was examined using logistic regression analysis, and odds ratios (ORs), and 95% confidence intervals (CIs) were calculated. The following variables were incorporated as covariates in the multivariate logistic regression analysis and multivariate analysis: age, male sex, diabetes mellitus, hypertension, and dyslipidemia. Statistical significance was set at *p* < .05. All statistical analyses were performed using SPSS for Windows version 20 (IBM Corp., Armonk, NY, USA).

### Ethics approval

All procedures performed in studies involving human participants were in accordance with the ethical standards of the institutional and/or national research committee and with the 1964 Helsinki Declaration and its later amendments or comparable ethical standards. The study was approved by the Research Ethics Committee of Saitama Sekishinkai Hospital (No. 2020–9) and Toho University Ohashi Medical Center (No. H20047).

### Consent to participate

The requirement for informed consent was waived because of the retrospective study design.

## Results

### Participants’ characteristics

Based on the inclusion and exclusion criteria, 3268 patients were enrolled in the study. Their mean age was 71 (range, 64–77) years, 72% were men, and 44% had diabetes mellitus. The median eGFR was 65 (range, 53–77) mL/min/1.73 m^2^. The lesion was observed in the LAD, RCA, and LCX in 56%, 28%, and 22% of the patients, respectively (Table 1). Online Resource 1 shows the characteristics of participants in CKD groups 1–5. Age, percentage of patients with diabetes mellitus, and hypertension increased in parallel with the CKD stage. On blood examination, non-HDL-C and Hb levels decreased significantly in parallel with the CKD stage increase, whereas BNP level increased gradually from CKD 1 to CKD 5.

**Table 1.** Patients’ characteristics.

No. of patients	3268
**Age, *y***	71 [64, 77]
**Male, *n* (%)**	2369 (72)
**Diabetes, *n* (%)**	1433 (44)
**Hypertension, *n* (%)**	2092 (64)
**Dyslipidemia, *n* (%)**	1708 (52)
**eGFR, mL/min/1.73 m^2^**	65 [53, 77]
**Coronary lesion**	
**RCA, *n* (%)**	914 (28)
**LMT, *n* (%)**	101 (3)
**LAD, *n* (%)**	1832 (56)
**LCX, *n* (%)**	733 (22)
**Number of lesions**	
**Single, n (%)**	2183 (67)
**Multiple (two or more), *n* (%)**	1085 (33)

Median [interquartile range].

eGFR: estimated glomerular filtration rate; RCA: right coronary artery; LMT: left main trunk; LAD: left anterior descending; LCX: left circumflex.

### CAD characteristics

Online Resource 2 shows the distribution of coronary lesions in each CKD stage. In CKD 1, the LAD was most commonly affected (57% of patients). The RCA was second, at 26%, but it increased to 31%, 38%, and 59% in CKD 3, 4, and 5, respectively. In CKD 5 patients, the RCA was the most commonly affected artery (59%), and LAD lesions accounted for 41% of the patients, while 17% had a lesion of the LCX. It should be noted that multiple lesions were present in 1085 cases (33.2%), of which 794 (24.3%) had two-branch lesions and 291 (8.9%) had three-branch lesions. This upward tendency was observed only for the RCA lesions.

### Association of coronary lesion with CKD stage

Logistic regression analysis showed that the ORs for an RCA lesion were 1.79 in CKD 4 and 4.21 in CKD 5, compared with CKD 1, after adjusting for traditional coronary risk factors such as age, sex, diabetes mellitus, and dyslipidemia (Table 2). For single lesions, ORs of 1.615 and 2.155 were identified in CKD 3 and 4, respectively (Online Resource 3). Propensity score matching was performed for patients with an RCA lesion, indicating an OR of 3.658 in CKD 5 with multiple lesions (Online Resource 4), and no significant associations were between LAD or LCX lesions and CKD stage (Online Resources 5 and 6).

**Table 2.** Association between coronary lesions and CKD stage.

RCA	Univariate		Multivariate^a^	
	OR (95% CI)	*p* Value	OR (95% CI)	*p* Value
**eGFR (mL/min/1.73 m^2^)**	0.99 (0.98–0.99)	<.001	0.99 (0.98–0.99)	<.001
**90 ≤ eGFR**	Reference		Reference	
**60 ≤ eGFR < 90**	0.94 (0.71–1.25)	.703	0.95 (0.71–1.26)	.748
**30 ≤ eGFR < 60**	1.24 (0.93–1.65)	.137	1.28 (0.94–1.73)	.105
**15 ≤ eGFR < 30**	1.70 (1.06–2.73)	.027	1.79 (1.10–2.94)	.019
**eGFR < 15**	3.96 (1.81–8.66)	.001	4.21 (1.90–9.29)	<.001

CKD: chronic kidney disease; RCA: right coronary artery; eGFR: estimated glomerular filtration rate; OR: odds ratio; CI: confidence interval.

^a^Adjusted for age, male sex, diabetes, hypertension, and dyslipidemia.

## Discussion

### Main findings

In this retrospective, cross-sectional study, the association between the location and/or distribution of coronary atherosclerosis lesions and CKD severity was investigated. Based on the analysis of 3268 consecutive unselected patients undergoing elective PCI, there were three impressive findings: (1) the prevalence of RCA lesions increased in advanced CKD patients, (2) this tendency was not observed for LAD and LCX lesions, and (3) after adjusting for traditional coronary risk factors, the ORs for RCA lesions in CKD 5 patients was approximately 4 compared to those with CKD 1. Although the mechanism remains unclear, the progression pattern clearly differs among the three major coronary arteries based on CKD severity.

### RCA lesions prevalent in CKD patients

In CKD 1, approximately 60% of the patients had a LAD lesion, which was the most common lesion at this stage. LCX and RCA lesions were observed in 20%–25% of patients. Almost the same distribution was observed in CKD 2 patients. These findings imply that an LCX lesion distribution is characteristic of the non-CKD population. This is in line with a previous report [8] that approximately 50% of non-CKD patients (i.e. 1636 consecutive Japanese patients with acute myocardial infarction), defined as those with eGFR >60 mL/min/1.73 m^2^, had LAD lesions. By contrast in patients with eGFR <60 mL/min/1.73 m^2^, the distribution of CAD changed from the LCX prevalent pattern to the RCA prevalent pattern with increased CKD stage; thus, 60% of CKD 5 patients had RCA lesions, more than those with LAD lesions (41%). On multiple logistic regression analysis, ORs of 1.7 and 4 were confirmed for CKD 4 and 5 patients, respectively, compared with CKD 1 patients after adjusting for traditional risk factors. This is supported by the evidence that approximately 42% of patients had RCA lesions, which was higher than CKD patients (35%) with LAD lesions [8]. Moreover, the prevalence of RCA lesions was higher than that of LAD lesions in 67 asymptomatic hemodialysis patients who volunteered for coronary angiography [12].

Although the present findings could have occurred by chance, to minimize selection bias and determine the specific effect of CKD on coronary atherosclerosis, patients with previous coronary revascularization therapy were excluded, and only the findings of angiography for the primary event were selected and analyzed. Therefore, the finding of an RCA prevalent distribution of CAD in advanced CKD patients is highly credible.

### Clinical implications

One must ask what an RCA prevalent distribution of CAD means in a clinical setting. A sinus or junctional bradycardic response in the setting of inferior myocardial infarction due to a responsible lesion in the proximal RCA is frequently observed. An early report indicated that 75% of patients with inferior myocardial infarction presented with sinus bradycardia within 15 min of infarction, as opposed to 15% of patients with anterior myocardial infarction [13]. However, an Australian group impressed us with the finding that sudden cardiac death in hemodialysis patients was caused by bradycardia and asystole, such as sudden cardiac arrest without ventricular arrhythmia [14]. A similar finding was confirmed by a Brazilian group, who showed a high frequency of fatal bradyarrhythmias on monitoring of loop recorders in 100 chronic hemodialysis patients [15]. In a study of predialysis diabetic CKD patients, bradyarrhythmia was more common than ventricular or supraventricular arrhythmias detected by loop recorder analysis [16]. Although this is only a hypothesis, the bradyarrhythmias observed in advanced CKD patients may be associated with the presence of coronary occlusive disease in the RCA. If this is true, the RCA lesion should be located in a proximal position. It is of great interest that there is a difference in the lesion position among the three coronary branches in CKD patients. Charytan et al. demonstrated that a more proximal lesion location was observed in CKD patients with myocardial infarction than in non-CKD patients [9]. When the major vessels were examined individually, patients with CKD stage 3 or higher had more proximal lesions in the RCA, but not in the LAD. Further studies are needed to determine the clinical implications of RCA prevalent lesion distribution in CKD patients. The mechanism of the RCA-prevalent lesion distribution in the uremic milieu is explored in Online Resource 7. The distribution of lesion location in patients with various stages of CKD can help clinicians inform patients about potential risks related to the condition, increasing patient and care-taker awareness with the potential to identify early signs and symptoms.

### Limitations

Although the sample size of this study was not very small, the retrospective, cross-sectional design does not permit a discussion of the causal effect of the uremic milieu on coronary atherosclerosis in the RCA. Due to the retrospective nature of our study, we were unable to extract data regarding specific branches of the RCA, which limits detailed analysis. Unfortunately, it was impossible to examine risk factors for coronary disease or CKD, such as serum phosphate, on RCA lesions because the results for such markers were not included in the database. Deterioration of renal function in advanced stages of CKD can lead to fears of contrast-enhanced nephropathy. Except for urgent cases, those with advanced stages of CKD, especially CKD5, have a low risk of affecting renal function from the use of contrast-enhanced coronary angiography, but it seems that the number of cases was small. It is possible that contrast medium was used in patients included in this study. As a result, it is possible that the number of cases is biased. Furthermore, as a cross-sectional study, we present real-world prevalence data for the studied institution and included time range. However, the lack of equal numbers across groups limits our statistical analysis of potential morphological and physiological variance between groups. Future studies should include longitudinal studies of demographically matched patients to validate observed differences across stages.

## Conclusion

In the patients analyzed in this study, the prevalence of RCA lesions, but not LAD or LCX lesions, significantly increased with increasing CKD stage. The pathophysiology of coronary atherosclerosis may differ according to location. Deterioration of renal function may affect the progression of atherosclerosis more in the RCA than in the LAD or LCX. While our study provides preliminary data to support these conclusions, further studies with more patients with stage 5 CKD using longitudinal designs and demographic matching across stages are required to validate these findings.

## Supplementary Material

Supplemental Material

Supplemental Material

Supplemental Material

Supplemental Material

Supplemental Material

Supplemental Material

Supplemental Material

## Data Availability

The data sets generated and/or analyzed during the current study are not publicly available but are available from the corresponding author on reasonable request.
