# Supplementary material for: Coronary artery lesion distribution in patients with chronic kidney disease undergoing percutaneous coronary intervention
Source: Ren Fail. 2022 Jul 8;44(1):1098–103. doi: 10.1080/0886022X.2022.2093748 (PMC9272943; doi:10.1080/0886022X.2022.2093748)
Supplement: Supplemental Material [file IRNF_A_2093748_SM3053.pdf]

Journal name: *Renal Failure*

Coronary artery lesion distribution in patients with chronic kidney disease

Naofumi Ikeda, Toshihide Hayashi, Shikou Gen, Nobuhiko Joki, Kazuhiko Aramaki

Corresponding Author:
Naofumi Ikeda
Department of Nephrology, Saitama Sekishinkai Hospital
2-37-20 Irumagawa, Sayama, Saiatama 350-1305, Japan
Tel.: +81-4-2953-6611 ; Fax: +81-4-2953-8040
E-mail: naofumi-ikeda@saitama-sekishinkai.org

Online Resource 2: Patients' characteristics by CKD stage

Variable	CKD stage					*P* value
	G1 (n = 300)	G2 (n = 1,708)	G3 (n = 1,128)	G4 (n = 103)	G5 (n = 29)	
**EF (%)**	60 [51, 66]	64 [55, 67]	62 [49, 67]	56 [46, 63]	53 [46, 63]	<0.001
**LVMI (g/m²)**	124 [111, 143]	125 [109, 144]	130 [112, 152]	152 [118, 168]	151 [136, 171]	<0.001
**RWT**	0.41 [0.37, 0.44]	0.41 [0.38, 0.45]	0.41 [0.38, 0.45]	0.40 [0.36, 0.45]	0.39 [0.35, 0.43]	0.430
**Coronary lesion, n (%)**						
**RCA**	79 (26)	432 (25)	347 (31)	39 (38)	17 (59)	<0.001
**LMT**	7 (2)	52 (3)	36 (3)	6 (6)	0 (0)	0.390
**LAD**	171 (57)	976 (57)	619 (55)	54 (52)	12 (41)	0.325
**LCX**	63 (21)	390 (23)	251 (22)	24 (23)	5 (17)	0.906
**Number of lesions, n (%)**						
**Single**	213 (71)	1,145 (67)	743 (66)	64 (62)	18 (62)	0.383
**Multiple (two or more)**	87 (29)	563 (33)	385 (34)	39 (38)	11 (38)	0.383

Median [interquartile range]
Note: CKD stages were defined by eGFR (G1, 90 ≤ eGFR; G2, 60 ≤ eGFR < 90; G3, 30 ≤ eGFR < 60; G4, 15 ≤ eGFR < 30; G5, eGFR < 15 mL/min/1.73 m^2).
CKD, chronic kidney disease; EF, ejection fraction; LVMI, left ventricular mass index; RWT, relative wall thickness; RCA, right coronary artery; LMT, left main trunk; LAD, left anterior descending; LCX, left circumflex.
