# Supplementary material for: Coronary artery lesion distribution in patients with chronic kidney disease undergoing percutaneous coronary intervention
Source: Ren Fail. 2022 Jul 8;44(1):1098–103. doi: 10.1080/0886022X.2022.2093748 (PMC9272943; doi:10.1080/0886022X.2022.2093748)
Supplement: Supplemental Material [file IRNF_A_2093748_SM4479.pdf]

**Journal name:** *Renal Failure*

## Coronary artery lesion distribution in patients with chronic kidney disease

Naofumi Ikeda, Toshihide Hayashi, Shikou Gen, Nobuhiko Joki, Kazuhiko Aramaki

Corresponding Author:
Naofumi Ikeda
Department of Nephrology, Saitama Sekishinkai Hospital
2-37-20 Irumagawa, Sayama, Saiatama 350-1305, Japan
Tel.: +81-4-2953-6611 ; Fax: +81-4-2953-8040
E-mail: naofumi-ikeda@saitama-sekishinkai.org

***Online Resource 7***

***Mechanism of the RCA prevalent lesion distribution in the uremic milieu***

The most important point is to clarify the mechanism by which RCA lesions progress in parallel with the development of a uremic milieu. Two potential mechanisms may be involved. One is that a combination of mechanical force with mineral bone disorder (MBD), which is one of the major CKD-specific complications, may play a role. Coronary hinge motion, a mechanical force on the coronary artery, is frequently observed in the RCA, which is a trigger for plaque rupture [1,2] or stent fracture [3]. Mechanical forces are known to have a significant effect on the initiation and/or progression of atherosclerosis [4]. Severe coronary artery atherosclerosis and calcification are often seen in advanced-stage CKD patients [5], and they have an influence as predictors of poor prognosis [6]. The prevalence of CKD patients with severe calcification with a coronary artery calcification score > 400 increases with CKD stage, because the serum phosphate level, the most important MBD marker and contributor to vascular calcification, increases with the CKD stage, especially after CKD 4 [7]. One possibility is that the combination of mechanical force with a high-phosphate condition may promote the progression of calcified atherosclerosis, especially in the RCA area. As reported, the presence of a calcified nodular lesion is associated with a larger hinge movement of the coronary artery (especially in the ostial or mid-RCA) in hemodialysis patients [8]. In addition, aortic valve calcification is induced by mechanical stretching with high-phosphate medium conditions in an animal model study [9]. These findings may support combination theory.

The second hypothesis is based on the field of experimental embryology. Arima et al. reported an intriguing result, published in a 2012 animal model study [10], in which neural crest cells from the preotic region migrate into the heart and differentiate into coronary artery smooth muscle cells. Neural crest cells primarily give rise to the craniofacial skeleton [11], which means that they have the potential to develop into bone. The main source of coronary smooth muscle cells is proepicardial mesenchymal cells [12]. Arima et al. demonstrated that preotic neural crest cells differentiate into coronary artery smooth muscle cells, but not all parts of the coronary artery. They are preferentially distributed in the orifice region,

including RCA. These findings imply that location heterogeneity may exist in the mechanism underlying the progression of coronary atherosclerosis. A recent report demonstrated that neural crest-derived smooth muscle cells are more prone to calcification than those of mesodermal origin [13].

Considering this evidence comprehensively, coronary atherosclerosis, including calcification in the RCA, especially at the ostial part, may progress more because of non-classical risks such as the uremic milieu than Framingham risks, whereas Framingham risks may be more important than non-classical risks in the LAD or LCX. However, to date, this is the only hypothesis.

**References**


1 Higuma T, Soeda T, Abe N, Yamada M, Yokoyama H, Shibutani S et al (2015) A combined optical coherence tomography and intravascular ultrasound study on plaque rupture, plaque erosion, and calcified nodule in patients with ST-segment elevation myocardial infarction: incidence, morphologic characteristics, and outcomes after percutaneous coronary intervention. JACC Cardiovasc Intv 8(9):1166-1176. https://doi.org/10.1016/j.jcin.2015.02.026

2 Mori H, Finn AV, Atkinson JB, Lutter C, Narula J, Virmani R (2016) Calcified nodule: an early and late cause of in-stent failure. JACC Cardiovasc Intv 9(13):e125-e126. https://doi.org/10.1016/j.jcin.2016.03.036

3 Omar A, Pendyala LK, Ormiston JA, Waksman R (2016) Review: stent fracture in the drug-eluting stent era. Cardiovasc Revasc Med 17(6):404-411. https://doi.org/10.1016/j.carrev.2016.06.002

4 Joki N, Kaname S, Hirakata M, Hori Y, Yamaguchi T, Fujita T, et al (2000) Tyrosine-kinase dependent TGF-beta and extracellular matrix expression by mechanical stretch in vascular smooth muscle cells. Hypertens Res 23(2):91-99. https://doi.org/10.1291/hypres.23.91

5 Kramer H, Toto R, Peshock R, Cooper R, Victor R (2005) Association between chronic kidney disease and coronary artery calcification: the Dallas Heart Study. J Am Soc Nephrol 16(2):507-513. https://doi.org/10.1681/ASN.2004070610

6 Joki N, Hase H, Takahashi Y, Ishikawa H, Nakamura R, Imamura Y et al (2003) Angiographical severity of coronary atherosclerosis predicts death in the first year of hemodialysis. Int Urol Nephrol 35(2):289-297. https://doi.org/10.1023/b:urol.0000020356.82724.37

7 Nakano C, Hamano T, Fujii N, Matsui I, Tomida K, Mikami S et al (2012) Combined use of vitamin D status and FGF23 for risk stratification of renal outcome. Clin J Am Soc Nephrol 7(5):810-819. https://doi.org/10.2215/CJN.08680811

8 Lee T, Mintz GS, Matsumura M, Zhang W, Cao Y, Usui E et al (2017) Prevalence, predictors, and clinical presentation of a calcified nodule as assessed by optical coherence tomography. JACC Cardiovasc Imaging 10(8):883-891. https://doi.org/10.1016/j.jcmg.2017.05.013

9 Balachandran K, Sucosky P, Jo H, Yoganathan AP (2010) Elevated cyclic stretch induces aortic valve calcification in a bone morphogenic protein-dependent manner. Am J Pathol 177(1):49-57. https://doi.org/10.2353/ajpath.2010.090631

10 Arima Y, Miyagawa-Tomita S, Maeda K, Asai R, Seya D, Minoux M et al (2012) Preotic neural crest cells contribute to coronary artery smooth muscle involving endothelin signalling. Nat Commun 3:1267. https://doi.org/10.1038/ncomms2258

11 Minoux M, Rijli FM (2010) Molecular mechanisms of cranial neural crest cell migration and patterning in craniofacial development. Development 137(16):2605-2621. https://doi.org/10.1242/dev.040048

12 Majesky MW, Dong XR, Regan JN, Hoglund VJ (2011) Vascular smooth muscle progenitor cells: building and repairing blood vessels. Circ Res 108(3):365-377. https://doi.org/10.1161/CIRCRESAHA.110.223800

13 Leroux-Berger M, Queguiner I, Maciel TT, Ho A, Relaix F, Kempf H (2011) Pathologic calcification of adult vascular smooth muscle cells differs on their crest or mesodermal embryonic origin. J Bone Miner Res 26(7):1543-1553. https://doi.org/10.1002/jbmr.382
