# Supplementary material for: Coronary artery lesion distribution in patients with chronic kidney disease undergoing percutaneous coronary intervention
Source: Ren Fail. 2022 Jul 8;44(1):1098–103. doi: 10.1080/0886022X.2022.2093748 (PMC9272943; doi:10.1080/0886022X.2022.2093748)
Supplement: Supplemental Material [file IRNF_A_2093748_SM4477.pdf]

Journal name: *Renal Failure*

Coronary artery lesion distribution in patients with chronic kidney disease

Naofumi Ikeda, Toshihide Hayashi, Shikou Gen, Nobuhiko Joki, Kazuhiko Aramaki

Corresponding Author:
Naofumi Ikeda
Department of Nephrology, Saitama Sekishinkai Hospital
2-37-20 Irumagawa, Sayama, Saiatama 350-1305, Japan
Tel.: +81-4-2953-6611 ; Fax: +81-4-2953-8040
E-mail: naofumi-ikeda@saitama-sekishinkai.org

Online Resource 6: Association between coronary lesions and CKD stage

LCX	Univariate		Multivariate *	
	**OR (95% CI)**	***P* value**	**OR (95% CI)**	***P* value**
**eGFR (mL/min/1.73m^2)**	0.99 (0.99-1.00)	0.696	1.00 (0.99-1.00)	0.608
**90 ≤ eGFR**	Reference		Reference	
**60 ≤ eGFR < 90**	1.11 (0.82-1.50)	0.484	1.05 (0.77-1.42)	0.734
**30 ≤ eGFR < 60**	1.07 (0.78-1.47)	0.642	0.95 (0.68-1.32)	0.769
**15 ≤ eGFR < 30**	1.14 (0.67-1.95)	0.624	0.98 (0.56-1.71)	0.957
**eGFR < 15**	0.78 (0.28-2.13)	0.634	0.68 (0.24-1.87)	0.461

CKD, chronic kidney disease; LCX, left circumflex; eGFR, estimated glomerular filtration rate; OR, odds ratio; CI, confidence interval.
*Adjusted for age, male sex, diabetes, hypertension, and dyslipidemia
