# Supplementary material for: Coronary artery lesion distribution in patients with chronic kidney disease undergoing percutaneous coronary intervention
Source: Ren Fail. 2022 Jul 8;44(1):1098–103. doi: 10.1080/0886022X.2022.2093748 (PMC9272943; doi:10.1080/0886022X.2022.2093748)
Supplement: Supplemental Material [file IRNF_A_2093748_SM4471.pdf]

Journal name: *Renal Failure*

Coronary artery lesion distribution in patients with chronic kidney disease

Naofumi Ikeda, Toshihide Hayashi, Shikou Gen, Nobuhiko Joki, Kazuhiko Aramaki

Corresponding Author:
Naofumi Ikeda
Department of Nephrology, Saitama Sekishinkai Hospital
2-37-20 Irumagawa, Sayama, Saiatama 350-1305, Japan
Tel.: +81-4-2953-6611 ; Fax: +81-4-2953-8040
E-mail: naofumi-ikeda@saitama-sekishinkai.org

Online Resource 5: Association between coronary lesions and CKD stage

**LAD**	**Univariate**		**Multivariate***	
	**OR (95% CI)**	***P* value**	**OR (95% CI)**	***P* value**
**eGFR (mL/min/1.73m^2)**	1.00 (1.00-1.00)	0.008	1.00 (1.00-1.00)	0.010
**90 ≤ eGFR**	Reference		Reference	
**60 ≤ eGFR < 90**	1.00 (0.78-1.28)	0.963	1.02 (0.79-1.31)	0.856
**30 ≤ eGFR < 60**	0.91 (0.71-1.18)	0.511	0.93 (0.71-1.22)	0.621
**15 ≤ eGFR < 30**	0.83 (0.53-1.30)	0.420	0.83 (0.52-1.32)	0.447
**eGFR < 15**	0.53 (0.24-1.15)	0.110	0.52 (0.24-1.14)	0.106

CKD, chronic kidney disease; RCA, right coronary artery; eGFR, estimated glomerular filtration rate; OR, odds ratio; CI, confidence interval.
*Adjusted for age, male sex, diabetes, hypertension, and dyslipidemia
