# Supplementary material for: Coronary artery lesion distribution in patients with chronic kidney disease undergoing percutaneous coronary intervention
Source: Ren Fail. 2022 Jul 8;44(1):1098–103. doi: 10.1080/0886022X.2022.2093748 (PMC9272943; doi:10.1080/0886022X.2022.2093748)
Supplement: Supplemental Material [file IRNF_A_2093748_SM4469.pdf]

Journal name: *Renal Failure*

Coronary artery lesion distribution in patients with chronic kidney disease

Naofumi Ikeda, Toshihide Hayashi, Shikou Gen, Nobuhiko Joki, Kazuhiko Aramaki

Corresponding Author:
Naofumi Ikeda
Department of Nephrology, Saitama Sekishinkai Hospital
2-37-20 Irumagawa, Sayama, Saiatama 350-1305, Japan
Tel.: +81-4-2953-6611 ; Fax: +81-4-2953-8040
E-mail: naofumi-ikeda@saitama-sekishinkai.org

Online Resource 4: Propensity score matching results

**RCA**	**OR (95% CI)**	***P* value**
**90 ≤ eGFR**	Reference	
**60 ≤ eGFR < 90**	0.775 （0.546-1.098）	0.152
**30 ≤ eGFR < 60**	1.052 （0.734-1.509）	0.781
**15 ≤ eGFR < 30**	1.343 （0.739-2.441）	0.334
**eGFR < 15**	3.658 （1.175-11.394）	0.025

RCA, right coronary artery; eGFR, estimated glomerular filtration rate; OR, odds ratio; CI, confidence interval.
*Adjusted for age, male sex, diabetes, hypertension, and dyslipidemia
