# Supplementary material for: Coronary artery lesion distribution in patients with chronic kidney disease undergoing percutaneous coronary intervention
Source: Ren Fail. 2022 Jul 8;44(1):1098–103. doi: 10.1080/0886022X.2022.2093748 (PMC9272943; doi:10.1080/0886022X.2022.2093748)
Supplement: Supplemental Material [file IRNF_A_2093748_SM4467.pdf]

Journal name: *Renal Failure*

Coronary artery lesion distribution in patients with chronic kidney disease

Naofumi Ikeda, Toshihide Hayashi, Shikou Gen, Nobuhiko Joki, Kazuhiko Aramaki

Corresponding Author:
Naofumi Ikeda
Department of Nephrology, Saitama Sekishinkai Hospital
2-37-20 Irumagawa, Sayama, Saiatama 350-1305, Japan
Tel.: +81-4-2953-6611 ; Fax: +81-4-2953-8040
E-mail: naofumi-ikeda@saitama-sekishinkai.org

Online Resource 3: Logistical regression analysis of single-branch lesions

RCA	OR (95% CI)	*P* value
**90 ≤ eGFR**	Reference	
**60 ≤ eGFR < 90**	1.158	0.423
**30 ≤ eGFR < 60**	1.615	0.011
**15 ≤ eGFR < 30**	2.155	0.013
**eGFR < 15**	3.073	0.026

RCA, right coronary artery; eGFR, estimated glomerular filtration rate; OR, odds ratio; CI, confidence interval.
*Adjusted for age, male sex, diabetes, hypertension, and dyslipidemia
