# Supplementary material for: Coronary artery lesion distribution in patients with chronic kidney disease undergoing percutaneous coronary intervention
Source: Ren Fail. 2022 Jul 8;44(1):1098–103. doi: 10.1080/0886022X.2022.2093748 (PMC9272943; doi:10.1080/0886022X.2022.2093748)
Supplement: Supplemental Material [file IRNF_A_2093748_SM4461.pdf]

Journal name: *Renal Failure*

Coronary artery lesion distribution in patients with chronic kidney disease

Naofumi Ikeda, Toshihide Hayashi, Shikou Gen, Nobuhiko Joki, Kazuhiko Aramaki

Corresponding Author:
Naofumi Ikeda
Department of Nephrology, Saitama Sekishinkai Hospital
2-37-20 Irumagawa, Sayama, Saiatama 350-1305, Japan
Tel.: +81-4-2953-6611 ; Fax: +81-4-2953-8040
E-mail: naofumi-ikeda@saitama-sekishinkai.org

Online Resource 1: Patients' characteristics in relation to CKD stage

Variable	CKD stage					*P* value
	**G1 (n = 300)**	**G2 (n = 1,708)**	**G3 (n = 1,128)**	**G4 (n = 103)**	**G5 (n = 29)**	
**Age (y)**	65 [56, 70]	69 [62, 74]	75 [69, 81]	78 [72, 85]	77 [68, 82]	<0.001
**Male, n (%)**	230 (77)	1,296 (76)	770 (68)	58 (56)	15 (52)	<0.001
**Diabetes, n (%)**	151 (50)	713 (42)	494 (44)	56 (54)	19 (66)	0.001
**Hypertension, n (%)**	155 (52)	1,035 (61)	788 (70)	89 (86)	25 (86)	<0.001
**Dyslipidemia, n (%)**	153 (51)	919 (54)	576 (51)	45 (44)	15 (52)	0.395
**BMI (kg/m^2)**	23.4 [21.2, 26.2]	23.9 [21.8, 25.9]	23.6 [21.5, 25.8]	23.0 [21.3, 25.3]	23.1 [20.7, 25.5]	0.194
**eGFR (mL/min/1.73 m^2)**	98 [94, 107]	71 [66, 78]	51 [44, 56]	26 [22, 28]	12 [9, 14]	<0.001
**HDL-C (mg/dL)**	48 [40, 57]	48 [40, 58]	46 [39, 56]	41 [33, 52]	46 [36, 51]	<0.001
**Non-HDL-C (mg/dL)**	151 [125, 187]	153 [127, 181]	145 [119, 174]	136 [112, 160]	109 [94, 131]	<0.001
**Hb (g/dL)**	14.6 [13.4, 15.7]	14.2 [13.2, 15.3]	13.5 [12.2, 14.7]	11.4 [10.1, 12.5]	10.9 [9.7, 12.0]	<0.001
**BNP (pg/dL)**	37 [16, 132]	37 [16, 92]	82 [26, 294]	384 [92, 814]	599 [258, 1,476]	<0.001

Median [interquartile range]
Note: CKD stages were defined by eGFR (G1, 90 ≤ eGFR; G2, 60 ≤ eGFR < 90; G3, 30 ≤ eGFR < 60; G4, 15 ≤ eGFR < 30; G5, eGFR < 15 mL/min/1.73 m^2).

CKD, chronic kidney disease; BMI, body mass index; eGFR, estimated glomerular filtration rate; HDL-C, high-density lipoprotein cholesterol; Hb, hemoglobin; BNP, B-type natriuretic peptide**.**
